# On the Influence of Apologies on the Likelihood of Lawsuits in Cases of Perceived Medical Negligence: Analysis of Archival and Experimental Data

**DOI:** 10.2196/77493

**Published:** 2025-10-14

**Authors:** Nelly Arbel-Groissman, Eran Dorfman, Elad Yom-Tov, Paul D Feigin, Anat Rafaeli

**Affiliations:** 1 Management & Organizations Department, Robert H. Smith School of Business University of Maryland College Park, MD United States; 2 Faculty of Data and Decision Sciences Technion – Israel Institute of Technology Haifa Israel; 3 Department of Computer Science Bar-Ilan University Ramat Gan Israel

**Keywords:** disappointing medical care, malpractice, medical errors, medical lawsuits, social media

## Abstract

**Background:**

Disappointing medical care (DMC) encompasses cases of medical failures, malpractice, or errors. Literature suggests that individuals’ perceptions of harm resulting from medical procedures influence their intention to seek legal recourse and that apologies may mitigate the inclination for legal action.

**Objective:**

Here, we aim to scrutinize and potentially challenge this prevailing notion.

**Methods:**

We conducted 4 studies using a dataset of social media posts detailing possible DMC incidents to which we linked a proxy for legal action, specifically, future posts related to legal action. Study 1 used a machine learning model to predict a proxy for an intent to file a lawsuit based on the content of 3815 posts. Two preregistered crowdsourcing studies (N=1115) assessed the impact of different apology types on intention-to-sue in 10 diverse medical scenarios with 4 apology conditions. Finally, study 4 aimed to test whether the predictors of legal intent identified in the crowdsourced studies and modeled as a function of case attributes can generalize to an actual subset of 165 Reddit (Reddit, Inc) posts.

**Results:**

Results show that apologies are rarely mentioned in descriptions of DMCs and that the descriptions of DMCs predict the proxy of legal action (area under the curve [AUC]=0.78). Crowdsourcing studies reinforce these findings: people agree on which cases are worthy of legal action (interclass agreement: 0.96 in study 1 and 0.71 in study 2), and our results demonstrate that physical and emotional damage are independently the strongest predictors of intention to file a lawsuit, together accounting for 43%-48% of model variance. Apologies are not statistically significant predictors for the intent to file a lawsuit (*P*>.05), both separately and in interaction with physical and emotional damage. A model developed in the crowdsourcing study and based on the attributes of cases, when applied to large-scale data, reached an AUC of 0.67-0.70. However, these attributes did not capture the entire range of behaviors, as a model that was based on the words in the cases reached a significantly higher AUC of 0.79.

**Conclusions:**

Text-based apologies appear to exert little influence on individuals’ intention to file a lawsuit in DMC cases, while physical and emotional damage are the primary motivators. This suggests that medical providers aiming to mitigate legal risks must explore alternative interventions beyond apologies.

## Introduction

A meta-analysis found that preventable patient harm occurs in 6% of patients across medical care settings, and 12% of such events led to severe harm, creating prolonged or permanent disability or death [1]. When such harms occur, and more generally, when patients or their relatives perceive the care they received as mediocre or otherwise unsatisfactory, they may choose to file a lawsuit against their medical providers. Accordingly, medical liability accounts for 2.4% of total health care spending in the United States [2].

One example of preventable medical lawsuits is medical malpractice lawsuits, which have an escalating trend and far-reaching implications, including high costs for physicians, medical systems, and consumers [3-5]. Malpractice lawsuits also have detrimental emotional and functional effects [6,7]. Despite their potential positive influence on increasing patient safety, a systematic review found no association between these phenomena in most studies reviewed [8]. Furthermore, there is a persistently high rate of dropped or dismissed claims [3], suggesting that medical care providers might be able to find other ways to mitigate the negative effects of lawsuits before they are filed.

A service failure perspective can offer valuable insights into understanding the dynamics of potential legal actions by introducing the principle of resource exchange. This principle posits that the magnitude of the gap in resource exchange created by a service failure correlates with people’s negative responses [9,10]. Specifically, the principle proposes that as the loss caused by a failure is perceived as larger, people are more likely to view the situation as inequitable and are less likely to be satisfied.

The service failure literature also proposes diverse strategies to navigate the aftermath of negative reactions to service failure and to temper customer reactions. Suggestions have been made for potential positive attenuating effects of refunds, discounts, upgrades, management involvement, and apologies [11,12]. We focus here on 1 recommended strategy—of extending a genuine apology—which is argued to be particularly potent [13,14]. Apologies, recognized for their capacity to foster reconciliation, satisfaction, and trust, have been explored in both service and social contexts [15-17], but have yet to be systematically examined in the medical arena. Hence, our goal here is to examine and establish whether apologies do offer health care providers an avenue for attenuating people’s drive to seek legal recourse [18,19].

The act of apologizing embodies acknowledgment of a transgression, which can be accompanied by expressions of remorse, regret, or empathy [20,21]. The multifaceted nature of an apology raises an open question of whether apologies can attenuate negative emotions, repair damaged relationships, and enhance overall satisfaction. Such effects have been shown in research in various contexts [14,15,22-25]. Available research implicitly suggests that apologies bear a power to transcend service interactions, resonating with the social dynamics of apologies to foster forgiveness, reduce punitive intentions, and facilitate conflict resolution [20,26,27]. Thus, the seemingly simple act of apologizing may offer health care providers a path to alleviate people’s emotional distress, improve strained patient-provider relationships, and possibly stop the escalation into legal actions following a case of disappointing medical care (DMC). However, there is limited research on the effects of apologies in health care. Within this limited research, some studies report a decrease in the likelihood of lawsuits when health care providers disclose incident details and offer apologies [28-30]. In contrast, others report mixed or no significant effect [31-33].

A study of the effect of apologies is complicated by the fact that lay people often find it difficult to identify medical errors. In some cases, what people perceive as unsatisfactory medical care or outcome is, in fact, the expected care or outcome. To capture the full picture, we therefore refer to lay people’s perceptions of medical malpractice as DMC. Among DMCs, there may be instances of medical failures, malpractice, or errors. Available literature suggests that perceptions of the harm caused by medical procedures shape people’s inclinations to pursue legal action [33]. Experimental studies demonstrated that greater severity of harm is associated with reduced patient satisfaction, inclination to change health care providers, and intention to file a lawsuit [34,35]. However, previous studies approached physical and emotional harm that a DMC causes as a uniform concept, whereas the 2 constructs suggest different dynamics. For example, the National Association of Insurance Commissioners in the United States differentiates between emotional injury, which is described as a temporary injury where no physical damage occurred, and physical injury, which ranges from insignificant injuries (where no delay in recovery occurs) to grave injury (which results in lifelong care or fatal prognosis) or death [36]. Moreover, previous studies examine a narrow range of predetermined harm, without fully capturing people’s assessments of the DMC situation and damage [28-31,33].

Although research and conventional wisdom purport that apologies may mitigate the likelihood of legal ramifications following medical errors and deficiencies, we endeavor to scrutinize and potentially refute this prevailing belief. Specifically, we aim to test whether greater physical or emotional damage increases the likelihood of a lawsuit, and whether apologies moderate the likelihood of a lawsuit.

## Methods

### Data

We used posts from medical and legal forums in Reddit, a social media platform, as our source data for DMC events (cases of medical failures, malpractice, or errors) where apologies might be influential. The medical forums we used are AskDocs, Dentistry, Askdentists, Doctors, and Medicine; in these forums we searched for posts containing the words “neglig,” “malprac,” “careles,” “error,” “fail,” “mistak,” “complain,” “wrongdo,” “incompetent,” “incorrect,” “injury,” and “fixable.” The terms were stemmed to retrieve all possible forms of the filtering words. We removed duplicate posts written by the same author with the same title or text, and posts containing fewer than 10 characters. The title and text of each post were then concatenated. Our dataset for analysis included 3815 posts posted between February 13, 2011, and January 26, 2023.

We also extracted all the comments for each post and, to maintain a representative dataset, selected only one comment per post based on the following criteria: (1) comment was a direct reply to the author, (2) comment was not by an automatic moderator comment, and (3) comment score assigned by Reddit was higher than a value of –100. Approximately 76% (2902/3815) of the posts in the dataset had a comment, allowing us to examine later whether comments from other users contribute significantly to the prediction of intention to file a lawsuit.

As a proxy for people’s inclination to file a lawsuit, we retrieved posts in legal forums (Ask_Lawyer and LegalAdvice) posted by authors who appeared in our DMC dataset. We filtered the legal content to include only posts that were posted after the author had first posted in the DMC dataset. We manually examined the posts written by the same author in both the DMC and legal subreddits, and retained only legal posts that discussed the same event and the specific medical incident we retrieved in the DMC data. Posts were tagged as indicating no intention to file a lawsuit if the author did not subsequently post in legal subreddits or if the content of the post in the legal subreddit did not refer to an incident described earlier in medical subreddits. This tagging process yielded a dataset with a total of 3815 posts, of which 3760 had no indication of intention to pursue a legal action, and 55 had a positive intention to file a lawsuit. We interpret posts on legal forums as showing an increased legal intent, rather than confirming a filing. We regard this high-precision data on the intention to file a lawsuit, because “intent to” behavior is widely recognized as a strong predictor of actual behavior [37]. Some people who intend to file a lawsuit may not post on legal forums, so the recall of these is likely lower; that is, some people who decided to file a lawsuit likely did not post their intention on social media.

To complement the social media analysis, we conducted several crowdsourcing experiments (see below), selecting 10 posts from the retrieved medical posts. We selected the tested posts by manually scanning the dataset in search of DMC posts that are brief (to maintain participant attention) and describe different degrees of damage. Two independent judges categorized posts regarding the degree of damage they described (low- or high-damage), and we selected 5 posts judged as describing high damage and 5 describing less severe damage. To each of these cases, for the purpose of the crowdsourcing studies, we artificially added text indicating an apology from the medical caregiver to create a manipulation of the apology variable. We specifically manipulated the added texts to create 4 conditions, based on 4 versions of apology, as follows: (1) remorse: an apology that expresses remorse, (2) responsibility: an apology that admits responsibility, (3) repair: an apology that offers to repair the situation, and (4) control: no apology or any added text.

All the study cases and apologies are provided in Multimedia Appendix 1. To control for alternative explanations, we briefly edited the texts, eliminated personal information (eg, age, sex, race, location, and prescriptions), expressions of feelings or questions, and revised the texts such that all appeared as referring to the writer’s mother or father.

### Ethical Considerations

The crowdsourcing studies described herein received full ethics approval from the Behavioral Sciences Research Ethics Committee of the Technion - Israel Institute of Technology (approval numbers 2022-098 and 2023-016). All procedures followed were in accordance with the ethical standards of the responsible institutional committee on human experimentation. Informed consent was obtained electronically from all participants in the crowdsourcing study prior to data collection. Participants were compensated based on survey length; those in Study 2 received £0.55, and those in Study 3 received £1.00. We attest to maintaining the privacy and confidentiality of all research subjects. Reddit author names are anonymous, and crowdsourced user identifiers were anonymized prior to analysis and publication; no personally identifying information is included in the manuscript. Lastly, the study protocols were preregistered on AsPredicted (#114542 and #148676).

### Study 1: Can the Language of DMC Social Media Descriptions Predict (a Social Media Proxy of) Intention to File a Lawsuit?

The purpose of study 1 was to establish a baseline for predicting the intention to file a lawsuit based on the content of DMC reports.

In this study we applied machine learning models to predict intention to file a lawsuit from features of the full data set of 3815 posts; we included responses to the posts as potential predictors and used the proxy of legal action as the predicted variable (comparing the 55 cases where we found a proxy of intention to pursue legal action to the rest of the cases where there was no such proxy). Specifically, we used the text of each post and the Reddit metrics of the post (ie, post score [a score shown by Reddit that is the number of upvotes minus the number of downvotes] and number of comments) as potential predictors of the intention to file a lawsuit. In a separate analysis, we added the text of comments as potential predictors. The text of each post (and later, separately, the comment) was represented by words and word pairs that appeared in more than 1% of the posts or comments. From those, we selected 300 attributes using a filter-based feature selection algorithm [38]. We trained a random forest model with 1000 trees and a depth constraint of 5 splits to predict intention to file a lawsuit (other models, including logistic regression, decision tree, and K-nearest neighbors, were also tested, and gave inferior results). To rigorously assess model performance and generalize results, we used stratified K-fold cross-validation with 10 folds. The area under the curve (AUC) was computed to quantify and compare predictive performance.

### Study 2: Crowdsourcing Study of Self-Reported Intention to File a Lawsuit in Response to DMC Scenarios

Study 2 was a between-subjects crowdsourcing experiment, in which we asked naive judges to report their reactions to a total of 40 different DMC situations (10 medical cases * 4 manipulated apology versions). To experimentally manipulate apologies, we inserted short apology texts into each DMC situation. The apology texts were single-sentence statements designed to isolate 3 key components of an apology: affective expression (remorse), admission of responsibility, and offer of corrective action (repair). Full DMC situations with manipulated apology versions are provided in Multimedia Appendix 1. Participants were recruited through Prolific Academic [39], asked to read each case and respond to a short survey of their assessment of the case (and attention checks), and were monetarily compensated for their participation. Each participant saw 4 situations in random order, and after reading each situation, was asked to indicate on a 7-point Likert scale (1) the extent of physical damage, (2) the extent of emotional damage, and (3) their intention to file a lawsuit (on an 11-point continuous scale).

Following data collection, the intention to file a lawsuit was modeled using linear regression as a function of reports of physical damage, emotional damage, and the experimental condition (apology version). As a control, we also created a model to assess the effects of the specific cases (the described situations) on the intention to file a lawsuit.

### Study 3: Crowdsourcing Study of Paired Comparison Choices and Intention to File a Lawsuit in Response to DMC Scenarios

Study 3 used the same DMC situations as study 2, and also used a between-subjects design, comparing 40 different situations in total (10 medical cases * 4 manipulated apology versions). However, study 3 assessed the intention to file a lawsuit using a different method. Specifically, in study 3, we asked participants to compare pairs of situations and choose in which of the 2 situations they would sue (rather than rating and responding to each of the individual situations). Using dedicated statistical analyses, we then extracted the effects of the different attributes of individual situations on the intention to file a lawsuit.

For the study 3 data collection, each situation was paired with all other situations with a different medical case, producing 720 pairs of situations that were compared across all participants. Participants were randomly assigned to 1 of 144 groups, with each group comprising 5 participants assigned to view and respond to the same 5 different pairs of situations (144 groups × 5 pairs=720 pairs). After reading each pair of situations, participants were asked to indicate which of the situations represents (1) greater physical damage, (2) greater emotional damage, (3) in which situation they were more likely to file a lawsuit, and (4) explain their choice.

Study 3 data were analyzed using linear regression based on a learning-to-rank framework that is designed to analyze choice-among-pairs data [40]. Details of this analysis are given in Multimedia Appendix 1. A robustness check using the Bradley-Terry model [41] is also given in Multimedia Appendix 1.

### Study 4: Can Models Developed From Crowdsourcing Studies Scale to Real-World Proxies for Intention to File a Lawsuit?

Study 4 is based on 165 cases from the full DMC data, selected such that some possibly contained an apology while the others did not. Cases of possible apology were identified by filtering posts to include the stem “apology,” excluding phrases that insinuate an apology of the author rather than the medical care provider (eg, “I apologize for a long post” or “apologies for multiple posts”). We excluded cases of “I apolog,” “my apology,” “I’ll apolog,” “apologies if,” “apologies for,” “apologies my,” “apologize if,” “apologize for,” or “apologize my” to capture apologies while minimizing false positives. This approach may not capture all apologies, since some are expressed without the explicit use of the “apolog” stem. This resulted in 51 cases. We also created a comparison set of posts by randomly selecting 114 cases that did not contain an apology, thus creating a subset of 165 cases.

These 165 posts and the 10 posts analyzed in studies 2 and 3 were examined by 2 independent judges and dummy-coded (yes or no) for the following 5 categorical variables: (1) severity of damage [42]: 1=death, 2=permanent damage, 3=temporary damage; (2) type of the medical event [42]: 1=diagnostic, 2=surgical, 3=obstetric, 4=treatment, 5=medication, and 6=other; (3) did the incident happen in the hospital? (4) is the damage caused visible? (5) whether the text included an apology and, if so, its type (remorse, responsibility, or repair).

Note that during modeling these variables were transformed to 11 variables using one-hot encoding, with “temporary” serving as the reference level for severity, and “obstetric” and “treatment” are both the references for the type of event, since they are highly correlated.

Using the data of studies 2 and 3, we created linear regression models to predict the physical damage and the emotional damage values provided by participants with the codes mentioned above. These models were then applied to the 165 cases (with and without a social media apology) to predict the emotional and physical damage associated with them. Then, using the models developed in studies 2 and 3 for predicting the intention to file a lawsuit from physical and emotional damage, as well as apology, we predicted the intention to file a lawsuit in the 165 cases and compared them to the proxy evidence for the intent to file a lawsuit (posting in the legal forum). The accuracy of these predictions was assessed using AUC.

## Results

### Study 1: Can the Language of DMC Social Media Descriptions Predict (a Social Media Proxy of) Intention to File a Lawsuit?

The random forest model based on the text of the posts achieved an AUC of 0.78. The same model, with the addition of the responses to the post, reached an AUC of 0.79. Receiver operating characteristic curves are shown in Multimedia Appendix 1. The difference between the AUC of the 2 models was not statistically significant (*P*>.05), indicating that the content of the responses did not add information on the intention to file a lawsuit.

Several robustness checks of analyses using the same methodology on different subsets of the data are reported in Multimedia Appendix 1, but did not convey any useful insight.

### Study 2: Crowdsourcing Study of Self-Reported Intention to File a Lawsuit in Response to DMC Scenarios

We recruited 400 US-based participants and excluded 5 who failed the attention check question, leaving an effective sample of n=395 (M_age_ 40.8, SD_age_ 13.13; 50.2% female). In total, 77% identified as White, 8% as Black, and 7% as Asian. Approximately 68% of the participants had completed at least a college degree, and the median household income was US $62,500 (IQR US $42,637-$116,098).

There was high interclass agreement (ICC) among participants (physical damage ICC=0.97, emotional damage ICC=0.96, and intention to file a lawsuit ICC=0.96).

Table 1 presents the full details of a regression model for predicting the intention to file a lawsuit against a given apology version in a situation, the extent of physical damage, and the extent of emotional damage. Table 1 also reports the moderation of apology on the relationship between physical and emotional damage and the intention to file a lawsuit. The overall model was statistically significant (*F*_11,1568_=130.8, *P*<.001), explaining 48% of the variance in the intention to file a lawsuit. While physical damage (B=0.76, SE 0.08, *P*<.001) and emotional damage (B=0.84, SE 0.09, *P*<.001) are shown to offer significant and distinct predictors of the intention to file a lawsuit, a notion not recognized at all in previous research; none of the apology conditions differed significantly from the control condition.

Specifically, all 3 apology versions did not significantly predict the intention to file a lawsuit compared to the control version, which was the reference level, remorse (B=0.27, SE 0.69, *P*=.70), responsibility (B=0.18, SE 0.71, *P*=.79), and repair (B=–0.47, SE 0.72, *P*=.52).

The interactions of emotional damage with remorse version (B=–0.06, SE 0.14, *P*=.67), responsibility version (B=–0.04, SE 0.14, *P*=.79), and repair version (B=0.13, SE 0.13, *P*=.35) were also not significant predictors. Similarly, apology did not moderate the relationship between physical damage and the intention to file a lawsuit, and the interactions between physical damage and remorse version (B=0.02, SE 0.12, *P*=.89), responsibility version (B=0.03, SE 0.14, *P*=.79), and repair version (B=–0.08, SE 0.12, *P*=.48) were not significant predictors. In short, we find no significant effects of any type of apology on intention to file a lawsuit.

**Table 1:** Linear model predicting intention to file a lawsuit in 2 crowdsourcing studies: study 2 (self-reported intent to file a lawsuit) and study 3 (paired comparison of intent to file a lawsuit). The model shows that only physical and emotional damage predict the intention to file a lawsuit.

Predictors	Study 2				Study 3		
	Estimates	SE	*P* value	Estimates		SE	*P* value
Intercept	–2.65	0.47	<.001	–0.02		0.01	.13
Physical damage	0.76	0.08	<.001	0.44		0.01	<.001
Emotional damage	0.84	0.09	<.001	0.31		0.01	<.001
Remorse	0.27	0.69	.70	0.02		0.03	.47
Repair	–0.47	0.73	.52	–0.00		0.03	.92
Responsibility	0.18	0.71	.79	0.03		0.03	.21
Physical damage × remorse	0.02	0.12	.89	0.02		0.03	.42
Physical damage × repair	–0.08	0.12	.48	0.00		0.03	.90
Physical damage × responsibility	0.03	0.11	.79	0.06		0.03	.04
Emotional damage × remorse	–0.06	0.14	.67	0.00		0.03	.87
Emotional damage × repair	0.13	0.13	.35	–0.02		0.03	.60
Emotional damage × responsibility	–0.04	0.14	.79	–0.03		0.03	.33

### Study 3: Crowdsourcing Study of Paired Comparison Choices and Intention to File a Lawsuit in Response to DMC Scenarios

We recruited 733 US-based participants and excluded 13 who failed the attention check question, leaving an effective sample of n=720 (M_age_ 41.3, SD_age_ 14.69; 54% female). In total, 80% identified as White, 11% as Black, and 4% as Asian. Approximately 63% of the participants had completed at least a college degree, and the median household income was US $63,401 (IQR US $36,416-$98,320). We found high ICC among participants (physical damage ICC=0.82, emotional damage ICC=0.80, and intention to file a lawsuit ICC=0.71).

Table 1 presents the full details of a regression model for predicting the intention to file a lawsuit given the apology versions, extent of physical and emotional damage, and the interaction of apology versions with the predictors. The overall model reported in Table 1 was statistically significant (*F*_11,3588_=244.6, *P*<.001), explaining 43% of the variance in the intention to file a lawsuit. Consistent with the findings observed in study 2, physical damage (B=0.44, SE 0.01, *P*<.001) and emotional damage (B=0.31, SE 0.01, *P*<.001) significantly and separately predict the intention to file a lawsuit. Similarly, all 3 apology versions did not significantly predict the intention to file a lawsuit compared to the control version, which was the reference level, remorse (B=0.02, SE 0.03, *P*=.47), responsibility (B=0.03, SE 0.03, *P=*.21), and repair (B=–0.00, SE 0.03, *P=*.92).

The interactions of emotional damage with remorse version (B=0.00, SE 0.03, *P=*.87), responsibility version (B=–0.03, SE 0.03, *P=*.33), and repair version (B=–0.02, SE 0.03, *P=*.60) were not significant. Similarly, an apology did not moderate the relationship between physical damage and intention to file a lawsuit.

In short, here as well, with a different methodology, there are no effects of apology.

As a final test, we assessed the effect of apologies in the individual test cases using a stepwise linear regression model where the independent variables were the apology version (with or without the inclusion of physical damage and emotional damage). We found only 1 case—case number 10, where the apology had a statistically significant association with intention to file a lawsuit. Case number 10 was tagged in study 4 as describing a diagnostic error, occurred at the hospital, and resulted in permanent damage.

### Study 4: Can Models Developed From Crowdsourcing Studies Scale to Real-World Proxies for Intention to File a Lawsuit?

Feature tagging showed satisfactory agreement (Cohen κ severity of damage=0.63, *P*<.001; Cohen κ type of medical error=0.61, *P*<.001; Cohen κ visibility=0.63, *P*<.001; Cohen κ hospital=0.71, *P*<.001; Cohen κ apology version=0.70, *P*<.001). The prediction of physical damage based on study 2 data was statistically significant (*F*_8,1571_=150.5, *P*<.001), explaining 43% of the variance in physical damage. The prediction of emotional damage was also statistically significant (*F*_8,1571_=117.3, *P*<.001), explaining 37% of the variance. Details of these models are provided in Multimedia Appendix 1.

The prediction of physical damage based on study 3 data was statistically significant (*F*_8,3591_=349, *P*<.001), explaining 44% of the variance in physical damage. The model predicting emotional damage was also statistically significant (*F*_8,3591_=335.3, *P*<.001), explaining 43% of the variance. Details of the models are provided in Multimedia Appendix 1.

Applying the models of the crowdsourcing data to the 165 study 3 cases achieved an AUC of 0.70 with the study 2 model and an AUC of 0.67 with the study 3 model (the difference is not statistically significant). Receiver operating characteristic curves are shown in Multimedia Appendix 1. These results show that the models developed in the crowdsourcing study can be applied to real-world (social media) settings. However, the attribute set we developed did not capture all information in the posts, since using the words of the posts themselves produced a higher AUC (0.79).

## Discussion

Medical liability accounts for 2.4% of total health care spending in the United States, a significant part of which is due to legal action by patients and their families. It has been suggested that apologies by medical staff could reduce the likelihood of such lawsuits. We therefore sought evidence for the multifaceted dynamics surrounding medical malpractice incidents, with a particular focus on the influence of apologies and the severity of damages on individuals’ decisions to pursue legal recourse.

A randomized controlled trial, the gold standard of experimentation, is complicated (and possibly unethical) to implement given our research question. However, prior research typically used vignettes that described a limited part of possible medical situations and relied extensively on self-reports of medical professionals regarding these limited vignettes [29,30,33]. There are well-known biases and limitations of self-report [43,44], which our study overcomes by applying 3 alternative methodological approaches to the study of the effects of apologies on intent to file a lawsuit.

In our first study, we analyzed the language of actual social media posts as data, using a proxy for intention to file a lawsuit based on postings about the same case in legal forums; we presume that such posts indicated a greater intention to file a lawsuit. We found that the presence of an apology in the described cases did not influence intention to file a lawsuit. At the same time, the language of the social media post was predictive of our proxy for an intention to file a lawsuit. Comments a post writer received to a post (including, presumably, opinions which discourage or encourage said lawsuit) did not add information to this prediction, implying that advice provided by strangers on social media, although medical professionals (as indicated by their comment flairs), did not significantly influence users’ intentions to file a lawsuit.

Our next 2 studies used crowdsourced data collection pertaining to DMC cases describing physical damage, emotional damage, and intent to file a lawsuit. Our study 2 tested 10 DMC cases (from original Reddit social media posts), to which we added a manipulation of different types of apologies. Our study 3 was similar in its data and setting to study 2, but asked participants to compare 2 cases and choose where they were more likely to file a lawsuit. In both these studies, we elicited the effects of each case through our modeling, and we did not find any effects of apologies.

In study 4, we integrated the multiple methods from our first 3 studies, demonstrating that laboratory-developed models can be applied to predict the real-world proxy of intent to file a lawsuit.

All our studies—though different in their design setup—show remarkably similar results: they demonstrate the significant effects of the severity of damage that a DMC creates, and they show no effect of an apology on the intention to file a lawsuit. Moreover, although we intentionally developed and experimentally compared 3 versions of expressing an apology—remorse, responsibility, and repair—to a control version, we found no significant effect of any of these versions on the intention to file a lawsuit. Considering arguments regarding the effects of apologies, we suggest that there may be individual scenarios where an apology could reduce the intention to file a lawsuit (eg, case number 10 in our data). However, we argue that it is illegitimate to generalize from these specific scenarios to claim an overall effect of apologies on the intention to file a lawsuit. That said, we must call for future work to investigate a larger set of cases than we could assess to identify the specific, yet rare, cases where apologies might have the ability to reduce legal claims.

In addition to demonstrating the lack of effect of apologies, our study 4 provided a novel demonstration that models developed in a laboratory setting can be applied to real-world data, where the proxy for behavior differed entirely from the behavior on which they were created. In our case, we demonstrated that posting in a legal forum can serve as a proxy for crowdsourcing participants’ intent to file a lawsuit. However, we again note that here as well, apologies did not have a significant effect on the intention to file a lawsuit.

We propose that other apology constructs, such as the expression of remorse (which conveys an affective component) or an offer to attempt to repair the damage (which conveys an action component) [45], can induce different cognitive and emotional processes, which we could not measure in this effort, yet may be effective in this context. Future research should delve into whether various apology constructs influence distinct emotions or cognitions and how they interact with the features of medical malpractice cases.

Research repeatedly suggests that people expect and desire to receive apologies, and that these expectations are frequently unmet, or that apologies provided do not align with expectations [46-49]. This line of research further complicates the question of “what is an apology?” As Risen and Gilovich [50] showed, whether apologies are spontaneous versus coerced makes a difference, as does whether people observe or are actual targets of an apology. Reported disconnects between expectations and the satisfaction derived from an apology could be attributed to these and other variations between different ways in which apologies can be expressed. Moreover, the contemporary prevalence of apologies in various contexts, wherein apologies have become a ubiquitous practice used by individuals and nations alike [51], may have led to elevated expectations and a heightened demand for receiving apologies. Paradoxically, however, the fact that apologies are now commonplace may diminish their symbolic significance, rendering the simple mention of an apology less satisfying and less effective in promoting forgiveness, and generating a need for more elaborate versions of an apology.

In line with this perspective, we propose that for apologies to wield a meaningful healing influence, they must be conspicuous and accompanied by additional actions. In their research on the failure of “apology laws” in reducing medical malpractice litigation, McMichael et al [52] advise physicians not to apologize without specific training. Their findings suggest that apology laws, intended to protect medical care providers from lawsuits, may increase the individual’s risk of medical malpractice liability. However, Communication-and-Resolution Programs are increasingly implemented across hospitals in the United States, focusing on transparency, emotional support, improvement of care quality, and proactively providing compensation to patients experiencing negligent care [53]. For instance, disclosure programs in academic medical centers have been successful in reducing the rates of compensation and lawsuits [54]. In line with our findings that brief textual apologies have a limited impact on intentions to file lawsuits, we suggest that a promising avenue for future research lies in examining the interactional and additive effects of providing apologies and other elements of these programs. Another potential outcome associated with apologies is increased patient satisfaction, which may lead to a lower likelihood of filing a lawsuit.

Our findings are subject to several limitations that should be noted. First, all our medical descriptions were derived from natural descriptions by Reddit users. While Reddit provides access to large-scale, naturalistic narratives of patient experiences, its user base may not fully represent the general US population nor, more widely, the experience of people worldwide. Reddit users tend to be younger and possibly more technically inclined, which could limit the generalizability of study 1 and study 4 [55]. Posting in a legal subreddit, which serves as our proxy for legal intent in study 1, captures public expressions of legal concern, not actual filings. Additionally, while this proxy likely has high precision, it may miss users who intend to file a lawsuit but do not post on legal Reddit forums, or overestimate intent in those who post without subsequent serious legal consequences. Furthermore, all participants were residents of the United States, thereby familiarizing them with the cultural and legal frameworks relevant to medical malpractice. However, considering that cultural norms may influence perceptions of apologies and the propensity to initiate legal proceedings, the findings may not be universally applicable to non-US or non-Western contexts.

Second, in studies 2 and 3, we used 10 short descriptions of medical cases, which may have influenced their responses. Although using experimental methods helps us understand and control for possible confounding factors, the distinction between making hypothetical decisions about a medical situation in an experiment and making actual decisions in real life might lessen the perceived seriousness and the practical implications of the decision to seek legal recourse. Moreover, we do test a significantly larger set of cases than past research tested, yet we acknowledge that the range of medical errors is vastly larger than these 10 cases. Thus, there may be cases where an apology would reduce the intention to file a lawsuit, which our data did not cover. Furthermore, we tested 3 apology conditions, and there may be other forms of apology that would have a significant effect. For example, there are aspects of apologies that our studies do not address, such as nonverbal behavior [56], attribution of responsibility [29,57], gender [58], communication medium, or the influence of other corrective actions [33]. Here as well, we call for future research to examine whether and how these aspects of the act of apologizing interact to shape its overall effectiveness, and whether and how they further enrich the question of what constitutes an apology.

Notwithstanding these limitations, we believe our work makes several significant statements. First, to the best of our knowledge, no previous research on apologies in the medical domain has used social media data. Our methods suggest that accessing such data can provide researchers and policy makers with valuable insights into how patients and their relatives perceive, seek advice on, or respond to medical errors [59-61]. Second, our use of crowdsourcing studies, combined with real-world proxies for intention to file a lawsuit, provides a closer approximation of people’s behavior than any previous research has offered. Finally, our results, which replicate across 3 samples and methods, contribute by suggesting that the levels of physical and emotional damage are separate and the strongest predictors of intention to file a lawsuit, while apologies by medical staff have a negligible effect on this intention. These findings should not be interpreted as evidence that apologies are never helpful. Rather, the types of apologies tested here were brief textual statements embedded in vignettes, and real-life apologies often include more complex elements, such as nonverbal cues [56] or follow-up actions [33], that may influence outcomes differently.

As the health care landscape continues to evolve, a comprehensive understanding of the interaction between patients, their family members, and their care providers remains essential for medical practitioners and legal professionals alike. This research offers a foundation upon which future studies can build to further elucidate the intricacies of this critical interaction.

## Appendix

Multimedia Appendix 1 All additional information for this paper.

## Abbreviations

AUC: area under the (receiver operating characteristic) curve
DMC: disappointing medical care
ICC: interclass agreement

## Data Availability

The datasets generated or analyzed during this study are available from the corresponding author on reasonable request.
