# Supplementary material for: On the Influence of Apologies on the Likelihood of Lawsuits in Cases of Perceived Medical Negligence: Analysis of Archival and Experimental Data
Source: J Med Internet Res. 2025 Oct 14;27:e77493. doi: 10.2196/77493 (PMC12569484; doi:10.2196/77493)
Supplement: Multimedia Appendix 1 [file jmir_v27i1e77493_app1.docx]

### Study 1, Receiver Operating Characteristic Curves

**Figure S1.** *ROC Curve of the Performance of Random Forest Classifier Using Features of 3,815 Posts.*


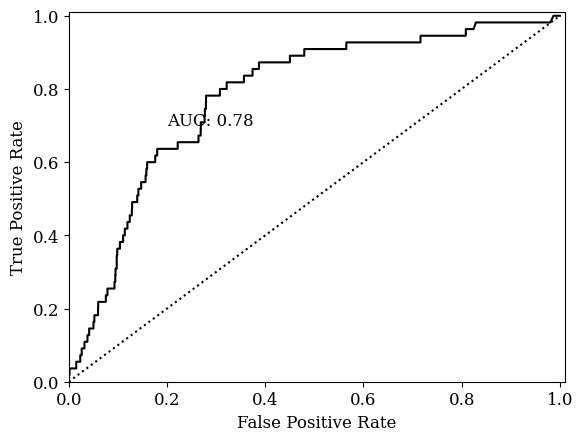


***Figure S2.*** *ROC Curve of the Performance of Random Forest Classifier Using Post and Comment Features of 3,815 Posts.*


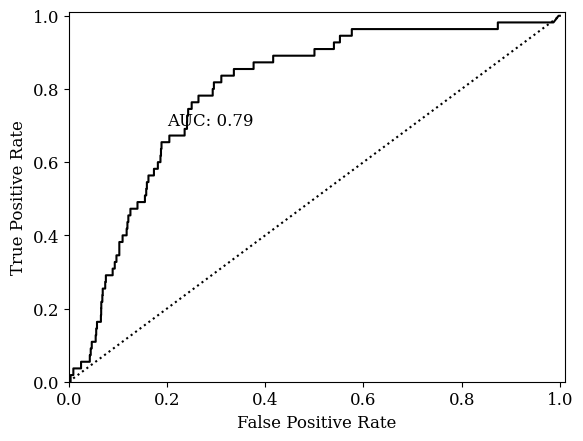


### Study 1, accuracy of prediction solely for cases in Study 4

Employing the same methodology of Study 1 (except for the use of Leave-One-Out cross-validation, due to the sample size) to the 165 cases analyzed in Study 4 reaches an AUC of 0.86 using the post text and 0.86 utilizing both text and comment (difference not statistically significant).

***Figure S3.*** *ROC Curve of the Performance of Random Forest Classifier Using Features of 165 Posts.*


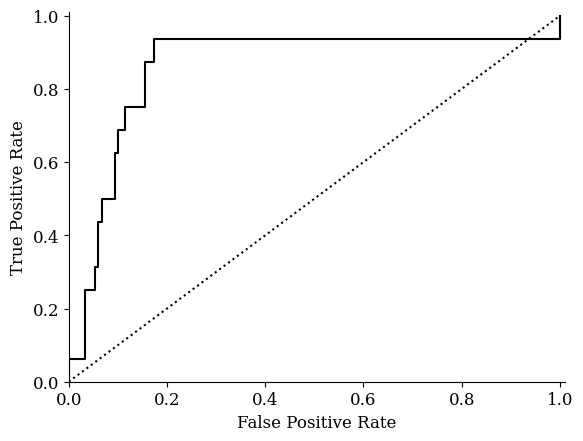


### Studies 2 & 3, List of Manipulated Cases

***Table S1.*** *Manipulated DOC Situations Tested in Studies 2 and 3.*

| Original Social Media Case | Apology Version | | | |
| --- | --- | --- | --- | --- |
|  | Remorse | Responsibility | Repair | Control |
| (1) My mother consumed rat poison because she saw a doctor (who might not even have a license) who told her that she won’t get better (most likely a lie) and she tried to k*** herself… | The doctor heard about the incident, called my mom to apologize, and said he feels terrible for what he said. Now she is OK. | The doctor heard about the incident, called my mom to apologize, and said that he is responsible for what happened. Now she is OK. | The doctor heard about the incident, called to my mom to apologize and asked her how he could repair their relationship. Now she is OK. | Now she is OK. |
| (2) My mother is about to die because the hospital put her feeding tube in wrong… | The doctor apologized and said she feels really awful; now she still has a bacterial infection from the misplaced feeding tube. She is in very bad condition and it doesn't look good from the doctor perspective. | The doctor have admitted to her error and apologized; now she still has a bacterial infection from the misplaced feeding tube. She is in very bad condition and it doesn't look good from the doctor perspective. | The doctor apologized and asked us what she could do to repair what happened; now she still has a bacterial infection from the misplaced feeding tube. She is in very bad condition and it doesn't look good from the doctor perspective. | Now she has a bacterial infection from the misplaced feeding tube. She is in very bad condition and it doesn't look good from the doctor perspective. |
| (3) My father receives monthly immunotherapy shots from an allergist to help with hayfever symptoms. This week, they accidentally gave him 2 times the proper dose of my injection… | They called after 10 minutes, apologized profusely, and said they feel very bad. As far as we can tell, he doesn't have any long-term effects from this. | They apologized profusely and let him know of their mistake after about 10 minutes. As far as we can tell, he doesn't have any long-term effects from this. | They called after about 10 minutes, apologized and offered several medications that they could give to make things right. As far as we can tell, he doesn't have any long-term effects from this. | As far as we can tell, he doesn't have any long-term effects from this. |
| (4) Hey, so my father recently has gotten his wisdom teeth out. While removing all 4 wisdom teeth his surgeon said he accidentally broke a root to his second molar which led it to have to be removed… | he apologized profusely and sounded genuinely remorseful. | he apologized and said he takes full responsibility for the harm he caused. | he apologized and offered to put in a bone graft which will allow my dad to put an implant for free in the future. |  |
| (5) My father had two fillings done on his two front teeth. After a month he is already noticing the filled teeth are getting darker and starting no longer to blend in. | My father emailed the dentist. The dentist replied and apologized saying he is very very sorry about this. | My father emailed the dentist. He replied saying he is sorry and takes full responsibility on the unwanted outcome. | My father emailed the dentist. He replied saying he is sorry and offered him few options to fix it. |  |
| (6) Roughly 3 years ago, my mother went in to have a kidney stone removed. Something went wrong, and an infection spread to her spine…  … Ever since then (about 27 months or so now) she's been bedridden and unable to stand, let alone walk. Her job's fired her (she worked for the hospital, in their IT dept), she's run out of insurance, and they're going to stop sending her money soon. | They apologized deeply and said they feel remorse for their actions. | They apologized deeply and said they take responsibility on the situation. | They apologized and said they'd try to fix that with a follow-up surgery. |  |
| (7) My father recently went to urgent care for chest pain he’d been experiencing for the last 2 days prior. This was his only symptom. He was given a Covid test (which was negative) and returned home. A few hours later, he died of a massive heart attack… | The urgent care staff said that they are truly sorry for our loss and feel very upset. | The urgent care staff apologized, and told us they take responsibility on their patients. | The urgent care staff apologized, and said that they will investigate the case. |  |
| (8) My father was in hospital battling cancer and had recently had a bone marrow transplant. He was not responding well, and there was talk of moving him to ICU but the doctors decided against it. Somehow, in the middle of the night, he aspirated his own vomit and died without any doctors or nurses noticing… | The medical staff said they were deeply sorry for his loss. | The medical staff said they were deeply sorry and feel responsible for his loss. | The medical staff were sorry for his loss and informed us that they plan to investigate the case. |  |
| (9) My mother was put under for an emergency C-Section. After they delivered my sister, they went to stitch her incision. In the middle of the process, the nurse's hand got in the way of needle and she ended up bleeding into the incision…  … Both parties had to be tested and everything came back okay. | The nurse and emergency room staff apologized and said they feel really bad about the incident. | The nurse and emergency room staff apologized and the nurse admitted her fault. | The nurse and emergency room staff apologized for the incident and offered reparation. |  |
| (10) My mother is currently in hospice dying of terminal brain cancer. The first hospital she went to when she fell ill sent her home after deeming her CT scan “unremarkable.” When she went to the hospital weeks later they found a huge brain tumor… | The first hospital' staff contacted and apologized, saying they are very sorry for what happened. | The first hospital' staff contacted her, apologized, and took responsibility for what happened. | The first hospital' staff contacted her, apologized, and offered to do whatever they could to repair the situation. |  |

##

### Study 3, Scale Items and Reliability Scores of Manipulation Check

***Table S2.*** *Scale Items and Reliability Scores of Manipulation Check.*

| Scale | Items | Cronbach's Alpha |
| --- | --- | --- |
| Remorse | The medical staff expressed feeling really sorry for what happened  The medical staff expressed feeling shame and remorse  The medical staff expressed remorse | 0.92 |
| Responsibility | The medical staff took responsibility for what happened  The medical staff acknowledged their behavior led to what happened  The medical staff took responsibility | 0.92 |
| Repair | The medical staff offered to repair what had happened  The medical staff offered to do something about what happened  The medical staff made an offer to repair | 0.94 |
| Apology | The medical staff apologized for what happened |  |
| Effectiveness | The medical staff response to the situation was effective |  |

### Study 3, Pretests

A series of pre-tests for agreement of participants regarding physical damage, emotional damage, and intention to file a lawsuit was conducted. The first and the second pre-tests were based on pairs of ten medical cases that were perceived by judges as presenting distinct seriousness levels. While the first pre-test that was conducted using Amazon Mechanical Turk (Mturk) platform ([www.mturk.com](https://www.mturk.com/)) showed low agreement, the second pre-test that was identical but conducted using Prolific Academic platform showed high agreement. Then we moved to a third pre-test, trying to ensure that there is a satisfactory agreement level between participants even when the cases that are being compared are not very different from each other in terms of seriousness. The third pre-test included all possible pairs of the ten medical cases.

Fifty participants were recruited through MTurk. To assure high quality data, the recruiting qualifications required were living in U.S., approvement rates of 98% and above, and more than 100 tasks approved in the platform. Each participant read one pair of cases out of ten pairs, in which one case was perceived as serious by the judges, and the other was perceived as less serious. The cases in each pair were presented in random order. Then, participants were required to choose which one of them caused greater physical damage to the patient, greater emotional damage to the patient, in which case a lawsuit is more justified and to answer an attention check. The pre-test resulted in low reliability rates (ICC _emotional_ = 0.53; ICC _physical_ = 0.34; ICC _sue_ = 0.45).

To check if the low agreement rates were due to controversial content described in the specific cases, a similar pre-test was conducted using another online panel, Prolific Academic platform, that has been shown to be more reliable for academic research (Douglas et al., 2023; Peer et al., 2022). Ten participants were recruited to the pre-test. To avoid the possibility that a case will be presented more than one time, participants were randomly assigned to groups, each group included five pairs and one attention check, that were presented in random order. In addition to the questions described above, participants were required to explain in a few words why they chose one case over the other regarding a lawsuit. The pre-test resulted in good reliability rates (ICC _emotional_ = 0.97; ICC _physical_ = 0.96; ICC _sue_ = 0.81), suggesting that the low agreement rates were not caused by the content, but by the raters.

Next, we ran an elaborate pre-test, including 45 pairs (all possible combinations of the ten cases used before). As before, participants were randomly assigned to one of nine groups, each group included five pairs and one attention check question, presented in a random order. Participants were asked to answer the same questions used in former pre-tests, regarding physical damage, emotional damage, intention to file a lawsuit, and to provide an explanation for their decision. 45 participants, located in the U.S. and whose first language is English, were recruited to this pre-study which provided good reliability rates (ICC _emotional_ = 0.92; ICC _physical_ = 0.92; ICC _sue_ = 0.87). The results showed that in general, there is an agreement between participants regarding the dependent and independent variables in these specific set of cases, allowing us to use the cases in the planned study.

Presence of Apology. One-way ANOVA showed significant difference in apology rates among the 4 apology versions (F(3, 187) = 60.55, *p* < .001). Tukey HSD corrected pairwise comparisons showed that remorse version (*M* = 3.98, *SD* = 1.12) was not perceived as more apologetic than repair version (*M* = 3.63, *SD* = 1.51, *p* =.43) or less than responsibility version (*M* = 4.28, *SD* = 1.01, *p* = .59), but was significantly more apologetic than the control version (*M* = 1.44, *SD* = 0.82, *p* < .001). The responsibility version was perceived significantly more apologetic than the repair version (*p* < .03) and more than the control version (*p* < .001). The repair version was perceived significantly more apologetic than the control version (*p* < .001). The results show that participants noticed the apologetic content in situations.

Perceived Offer for Reparation. Next, one-way ANOVA showed significant difference in repair rates between the versions (*F*(3, 187) = 42.09, *p* < .001). Tukey HSD corrected pairwise comparisons showed that repair version (*M* = 3.69, *SD* = 1.21) was rated significantly higher in repair scale than responsibility (*M* = 2.13, *SD* = 1.17, *p* < .001), remorse (*M* = 1.60, *SD* = 0.94, *p* < .001), and control (*M* = 1.55, *SD* = 0.93, *p* < .001). Remorse version was not rated significantly different than responsibility version (*p* = .08) or than control version (*p* =.99), while responsibility version received significantly higher repair rates than control version (*p* < .04).

Perceived Admission of Responsibility. One-way ANOVA showed significant difference in responsibility rates between the apology versions (*F*(3, 187) = 47.89, *p* < .001). Tukey HSD corrected pairwise comparisons showed that responsibility version (*M* = 3.96, *SD* = 1.05) was rated significantly higher than remorse version (*M* = 3.04, *SD* = 1.37, *p* = .001) and control version (*M* = 1.60, *SD* = 1.01, *p* < .001), but not than repair version (*M* = 3.38, *SD* = 1.31, *p* < .09). The remorse version was rated significantly higher in responsibility scale than control version (*p* = .001), but not different than repair version (*p* = .51). Repair version received significantly higher rates on responsibility scale compared to control (*p* < .001).

Perceived Expression of Remorse. Finally, one-way ANOVA showed significant difference in remorse rates between the apology versions (*F*(3,187) = 51.42, *p* < .001). Tukey HSD corrected pairwise comparisons showed that remorse version (*M* = 3.68, *SD* = 1.09) was rated significantly higher in remorse scale than control version (*M* = 1.45, *SD* = 0.81, *p* < .001) but not than responsibility (*M* = 3.82, *SD* = 1.08, *p* = .91) or repair versions (*M* = 3.26, *SD* = 1.20, *p* = .21). Responsibility version received significantly higher remorse rates than repair (*p* = .05) and control versions (*p* < .001). Repair version was rated significantly higher than control version (*p* < .001). Overall, these results show that participants distinguish between the apology versions, especially between control version to other three apology versions.

Effectiveness of Response. The manipulation check included an exploratory item that asked participants to rate the perceived effectiveness of each version ("The medical staff response to the situation was effective"). A one-way ANOVA showed a significant difference between the versions (*F*(3,187) = 14.90, *p* < .001). Tukey HSD corrected pairwise comparisons showed that control version (*M* = 1.71, *SD* = 1.11) was perceived significantly less effective than responsibility version (*M* = 2.51, *SD* = 1.43, *p* =.02) and repair version (*M* = 3.02, *SD* = 1.51, *p* < .001), but not from remorse version (*M* = 2.13, *SD* = 1.33, *p* = .43). Repair version was rated as the most effective response, significantly higher than remorse (*p* = .01) but not than responsibility (*p* = .26). Responsibility was not significantly more effective response than remorse (*p* < .51). This result suggests that remorse and responsibility, which are the most known constructs of apology in the literature (Robbennolt, 2003), are not necessarily perceived as the most effective responses after an incident occurred, compared to an offer to repair the situation.

***Table S3.*** *Descriptive Statistics of Scales by Apology Versions.*

| Apology Version | | Remorse | | Responsibility | | Repair | | Apology | | Effectiveness | |
| --- | --- | --- | --- | --- | --- | --- | --- | --- | --- | --- | --- |
|  | *N* | *M* | *SD* | *M* | *SD* | *M* | *SD* | *M* | *SD* | *M* | *SD* |
| Remorse | 48 | 3.68 | 1.09 | 3.04 | 1.37 | 1.60 | 0.94 | 3.98 | 1.12 | 2.13 | 1.33 |
| Responsibility | 47 | 3.82 | 1.08 | 3.96 | 1.05 | 2.13 | 1.17 | 4.28 | 1.01 | 2.51 | 1.43 |
| Repair | 48 | 3.26 | 1.20 | 3.38 | 1.31 | 3.69 | 1.21 | 3.63 | 1.51 | 3.02 | 1.51 |
| Control | 48 | 1.45 | 0.81 | 1.60 | 1.01 | 1.55 | 0.93 | 1.44 | 0.82 | 1.71 | 1.11 |

### Study 3, Analysis of paired selection data

The method developed by Joachims utilizes comparative judgments to establish rankings, and this approach was applied to assess user preferences between pairs of web pages displayed on a search results page (e.g., Google search). This approach serves as a foundation for learning about the ranking of web pages according to their specific parameters. Here, we apply this method to use participant choices between two alternative situations as data for identifying whether physical damage, emotional damage, apology versions, and the interactions between them predict the individual’s intention to file a lawsuit.

Let the level of intention of filing a lawsuit for a particular situation be:

Sue = f(X)

Where f(X) is a function of independent parameters ${\{x_{i}\}}_{i=1}^{N}$ of the situation, and f an aggregating function. Here we will assume that f is a linear function of the parameters. In our experimentation, we obtained the preference for suing for one situation over another, that is, if Sue1 and Sue2 are the intention to file a lawsuit for situation 1 and 2 respectively, the preferences are:

Sue2 - Sue1 = f(X(2)) – f(X(1))

Where X(1), X(2) are the independent parameters of situations 1 and 2, respectively. Denoting by bi the coefficients of the aggregating function f, and since f is linear:

Sue2 - Sue1 = f(X(2)) – f(X(1)) = $\sum_{i=1}^{N} b_{i} ^{\circ} \left( x_{i}^{\left( 2 \right)}-x_{i}^{\left( 1 \right)} \right)$ = $\sum_{i=1}^{N} b_{i} ^{\circ} \delta_{i}$

Where $\delta_{i}=\left( x_{i}^{\left( 2 \right)}-x_{i}^{\left( 1 \right)} \right).$

If the independent parameters for each situation are known, the difference among them can be calculated directly. Alternatively, when crowdsourcing workers choose an independent parameter by indicating preferences among pairs of situations (e.g., which situation caused greater physical damage), they are providing the difference between these parameters for a pair of situations (e.g., $\delta_{i}$).

Once the differences between the parameters of situations are given, equation (3) can be solved (e.g., its parameters b can be estimated) via linear regression. Note that, once the regression coefficients are estimated, they can be applied to a single situation through equation (1). The values of physical and emotional damage, as well as intention to file a lawsuit, were collected from participants and are relative to the specific pairs of situations. Thus, the values of these variables were coded as (-1) when the first situation in a pair was indicated and (+1) when the second situation in the pair was indicated. In contrast, the apology versions were manipulated and not directly indicated. Therefore, the two apology versions associated with each pair needed to be translated into relative variables for model use.
The processing of apology versions was done as follows: We identified the position of each situation in a pair as either the 1st or 2nd. For each position, we defined four dummy variables, each representing one of the four apology versions (“remorse_1”, “responsibility_1”, “repair_1”, “control_1” for the first position; and similar variables for the second position). Then, to create relative variables for each pair, we created four additional dummy variables ($\delta_{i,4}$) representing the differences between apology versions within each pair (“remorse”, “responsibility”, “repair”, “control”). This was achieved by subtracting the value of the dummy variable of the 2nd situation from the value of the dummy variable of the 1st situation for each apology version. This approach yielded a relative value representing the apology versions of each situation in a pair. A difference dummy variable ($\delta_{i,4}$) value of +1 indicated assignment of the 2nd situation to that apology version (while the 1st situation was not assigned), value of -1 represented the reverse (1st situation assigned to this apology version while the 2nd situation not assigned), and 0 represented no difference between the apology versions of the situations in the pair. Once all these variables are calculated, a linear regression is performed to assess the relative influence of physical damage and emotional damage as linear predictors of the intention to file a lawsuit, and of the apology version as a moderator of this linear effect.

Study 3, Experiment results

Table S4 summarizes means, standard deviations, and intercorrelations among all the study variables. Below, we report the two sets of analyses that we conducted to test the study hypotheses.

***Table S4.*** *Means, Standard Deviations, and Correlations with Confidence Intervals.*

| Variable | *M* | *SD* | 1 | 2 | 3 | 4 |
| --- | --- | --- | --- | --- | --- | --- |
| 1. Intention to file a lawsuit | 0.04 | 1.00 |  |  |  |  |
| 2.Emotional damage | 0.10 | 1.00 | .53** |  |  |  |
|  |  |  | [.51, .56] |  |  |  |
| 3.Physical damage | 0.08 | 1.00 | .60** | .50** |  |  |
|  |  |  | [.57, .62] | [.48, .53] |  |  |
| 4. Age | 41.30 | 14.69 | .03* | -.00 | .01 |  |
|  |  |  | [.00, .07] | [-.04, .03] | [-.02, .04] |  |
| 5. Sex^a^ | 0.47 | 0.54 | -.04* | .01 | -.02 | -.08** |
|  |  |  | [-.07,-.00] | [-.03, .04] | [-.05, .02] | [-.11, .04] |

*Note.* N=3,600 observations by 720 participants. Values in square brackets indicate the 95% confidence interval for each correlation.

^a^ 0 = male and 1 = female.

* *p* < .05, ** *p* < .01.

### Study 3, Bradley-Terry Test: What is The Level of “Success” of Cases in Terms of Intention to File a Lawsuit?

Following Bradley & Terry (1952), we used what is known as the “Bradley-Terry model” and considered a participant’s indication of one situation rather than another as justifying a malpractice lawsuit as a 'success'. We then used a software package (Turner & Firth, 2012) to predict the success of a situation relative to another situation. The Bradley-Terry model is a probability model for paired evaluations, assuming that the probability of choosing one situation over another is proportional to its relative strength. Thus, for this method, we coded in each comparison (720 pairs of situations * 5 observations per pair) the situation indicated as more justifiable for a lawsuit (representing greater physical damage, emotional damage) as a winner, and the other version as a loser. While the coding of participants’ preferences for malpractice lawsuit served us as an initial check of our hypotheses, the coding of physical damage and emotional damage served as a test for an alternative explanation regarding the confounding effect of apology versions on the variables in question.

To run the *Bradley-Terry model* the data was processed, using the standard model and a maximum likelihood fit. The coefficients produced by the model (*B* ˆ _i_) are the model estimates compared to a reference level (*B* ˆ _0_ = 0). We analyzed the relative strength of each situation as the sum of the effect of the case and the effect of the apology version in an additive model with no interaction, regarding three outcomes – physical damage, emotional damage, and intention to file a lawsuit. The first two models, which were conducted in order to make sure that apology versions do not affect participants’ perception of the variables in question, showed no significant effects for apology versions on physical damage and emotional damage, and are reported fully below. Table S5 reports the estimates for all cases compared to case number 10, which was randomly selected as a reference point, and all apology versions are compared to control version as a reference level.

***Table S5.*** *Bradley Terry Model Predicting Intention to File a Lawsuit with Case and Apology Version.*

| Predictors | Estimates | *SE* | *p* |
| --- | --- | --- | --- |
| **Fixed Effects** |  |  |  |
| Case 1 | -0.74^***^ | 0.12 | **<.001** |
| Case 2 | 1.42 ^***^ | 0.13 | **<.001** |
| Case 3 | -1.36 ^***^ | 0.12 | **<.001** |
| Case 4 | -1.03^***^ | 0.12 | **<.001** |
| Case 5 | -1.69^***^ | 0.13 | **<.001** |
| Case 6 | 1.01^***^ | 0.13 | **<.001** |
| Case 7 | 0.57^***^ | 0.12 | **<.001** |
| Case 8 | 0.81^***^ | 0.12 | **<.001** |
| Case 9 | -1.34^***^ | 0.12 | **<.001** |
| Case 10 |  |  |  |
| Remorse | 0.14 | 0.08 | .10 |
| Responsibility | 0.14 | 0.08 | .09 |
| Repair | 0.07 | 0.08 | .37 |
| Control |  |  |  |
| **Random Effects** |  |  |  |
| Version | 0.03 | 0.16 | .86 |
| **** p < .001* | | | |

Figure S4 shows the probability estimates of each version using the quasi-likelihood approach, which considers the non-independence of the outcomes, to provide more accurate estimates of the standard errors. The results, as can be seen in Table S5 and Figure S4, show that all cases were significantly different from case 10 in their strength. Cases 2, 6, 7 and 8 had a significantly higher probability of being selected as justifying a lawsuit relative to case 10. While cases 1, 3, 4, 5 and 9 had significantly lower probability. These results suggest that different medical cases vary in their likelihood to affect the intent to file a lawsuit.

These results do not show any significant effects of apology on the intention to file a lawsuit. Across all situations there was no statistically different effect for apology versions on intention to file a lawsuit compared to the control version. The figure shows that estimates of intention to file a lawsuit vary between cases, but the apology versions in each case create small variations.

***Figure S4.*** *Intention to File a Lawsuit Estimates – Two Factor Model (Case & Apology Version).*

*
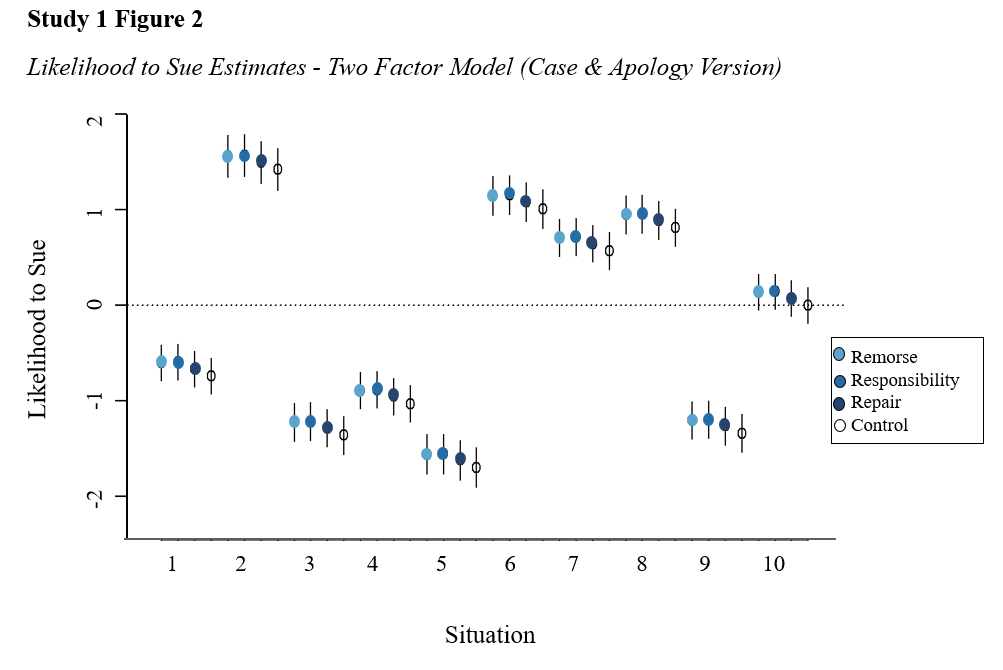
*

***Table S6.*** *Predicting Physical Damage and Emotional Damage by Case Features – Study 3.*

|  | | | | | | |
| --- | --- | --- | --- | --- | --- | --- |
|  | **Physical Damage** | | | **Emotional Damage** | | |
| Predictors | Estimates | *SE* | *p* | Estimates | *SE* | *p* |
| Intercept | -0.11 ^***^ | 0.02 | **<.001** | -0.01 | 0.02 | .46 |
| Severity of Damage | |  |  |  |  |  |
| Temporary |  |  |  |  |  |  |
| Permanent | 0.78 ^***^ | 0.04 | **<.001** | 0.67 ^***^ | 0.04 | **<.001** |
| Death | 1.16 ^***^ | 0.05 | **<.001** | 0.63 ^***^ | 0.05 | **<.001** |
| Type of Medical Adverse Event | | |  |  |  |  |
| Obstetric |  |  |  |  |  |  |
| Treatment |  |  |  |  |  |  |
| Diagnostic | -0.14 ^***^ | 0.03 | **<.001** | -0.01 | 0.03 | .70 |
| Surgical | 0.29 ^***^ | 0.03 | **<.001** | 0.06 ^*^ | 0.03 | **.02** |
| Medication | -0.25 ^***^ | 0.04 | **<.001** | -0.32 ^***^ | 0.04 | **<.001** |
| Other | 0.91 ^***^ | 0.05 | **<.001** | 1.55 ^***^ | 0.05 | **<.001** |
| Hospital | 0.74 ^***^ | 0.03 | **<.001** | 0.92 ^***^ | 0.03 | **<.001** |
| Visibility | -0.07 ^**^ | 0.03 | **<.01** | 0.14 ^***^ | 0.03 | **<.001** |
| *Note.* Temporary damage, treatment and obstetric medical adverse events variables were not estimable due to multicollinearity with other predictors and were omitted from the models.  ** p < .05, *** p < .001* | | | | | | |

***Table S7.*** *Predicting Physical Damage and Emotional Damage by Case Features – Study 2.*

|  | **Physical Damage** | | | **Emotional Damage** | | |
| --- | --- | --- | --- | --- | --- | --- |
| *Predictors* | *Estimates* | *SE* | *p* | *Estimates* | *SE* | *p* |
| Intercept | 3.02 ^***^ | 0.14 | **<.01** | 3.48 ^***^ | 0.12 | **<.01** |
| Severity of Damage | | | | | | |
| Temporary | | | | | | |
| Permanent | 2.65 ^***^ | 0.13 | **<.01** | 1.53 ^***^ | 0.12 | **<.01** |
| Death | 3.34 ^***^ | 0.17 | **<.01** | 1.56 ^***^ | 0.15 | **<.01** |
| Type of Medical Adverse Event | | | | | | |
| Obstetric | | | | | | |
| Treatment | | | | | | |
| Diagnostic | -0.71 ^***^ | 0.10 | **<.01** | -0.19 ^*^ | 0.09 | **.03** |
| Surgical | 0.66 ^***^ | 0.10 | **<.01** | -0.13 | 0.09 | .13 |
| Medication | -0.40 ^**^ | 0.14 | **.01** | -0.81 ^***^ | 0.13 | **<.01** |
| Other | 2.87 ^***^ | 0.17 | **<.01** | 2.92 ^***^ | 0.15 | **<.01** |
| Hospital | 0.80 ^***^ | 0.10 | **<.01** | 1.56 ^***^ | 0.09 | **<.01** |
| Visibility | -0.41 ^***^ | 0.10 | **<.01** | 0.10 | 0.09 | .26 |
| *Note.* Temporary damage, treatment and obstetric medical adverse events variables were not estimable due to multicollinearity with other predictors and were omitted from the models.  ** p < .05, *** p < .001* | | | | | | |

### Study 3, Data Quality Check

According to our hypotheses, the degree of physical damage and emotional damage of cases interacts with apology versions and affects the intention to file a lawsuit for malpractice. However, the study design is based on participants’ evaluations of the degree of physical damage and emotional damage of situations. Participants indicate which situation represents greater physical (emotional) damage after reading pairs of cases coupled with apology versions, which might unintentionally bias their perceptions.

To check if apology versions interfere in the evaluations of physical damage and emotional damage, we ran two Bradly-Terry models including cases and apology versions in an additive model as predictors. Since both models are based on the same data points and predictors, which raises the probability of making Type 1 error, we used Bonferroni correction. Thus, the adjusted alpha level we set was 0.025. Since in this situation we were interested in the overall effect of apologies and cases, rather than the effects of each case or each apology version, after running both models, we performed a Chi square test as a likelihood ratio test. Assessing the effects of cases and apology versions on emotional damage, cases were found significant ꭓ^2^(9, N = 3,600) = 836.00, *p* < .001; while apology versions were not significant ꭓ^2^(3, N = 3,600) = 8.45, *p* = .04. Assessing the effects of cases and apology versions on physical damage, cases were found significant ꭓ^2^(9, N = 3,600) = 986.10, *p* < .001; while apology versions were again not significant ꭓ^2^(3, N = 3,600) = 1.34, *p* = .72. Both tests showed significant effect for cases on the variables in question, and found no significant effect for apology versions, meaning that the manipulation of apology did not affect the perception of physical damage and emotional damage in situations. These results allowed us to move further in our analysis.

### Studies 2 and 3, Detailed Tables of Linear and Mixed Effects Models Performed in Hypotheses Testing

The data of Study 2 and 3 comprised of sets of ratings nested within an individual participant. This nested data violates the assumption of independence, so the analyses were conducted using multilevel linear modeling (MLM) of a 2-level model: Level 1 units are the measured variables and Level 2 units are participants' random effects. To test the hypothesized relationships, the seriousness condition, the apology version, and their interaction effect on the intention to file a lawsuit were included in the multilevel analyses along with participants identifiers as markers. All model estimations were conducted with Rstudio Statistical Software (RStudio Team, 2021).

**Study 2**:

We determined the appropriateness of the model by comparing Model 2a, a model that included random intercepts for each participant (to account for the nested design), with Model 1a, a general linear model. The linear-mixed effects model had the best fit to the data (ꭓ2 (1) = 67.80, p < .001), meaning that in this study, participants’ tendencies to sue for malpractice influenced the results.

***Table S8.*** *Detailed model parameters, Study 2.*

|  | **Model 1a: Linear Model** | | | **Model 2a: Mixed Effects Model** | | |
| --- | --- | --- | --- | --- | --- | --- |
| *Predictors* | *Estimates* | *SE* | *p* | *Estimates* | *SE* | *p* |
| Intercept | -2.65 ^***^ | 0.47 | **<.001** | -2.70 ^***^ | 0.46 | **<.001** |
| Physical Damage | 0.76 ^***^ | 0.08 | **<.001** | 0.69 ^***^ | 0.08 | **<.001** |
| Emotional Damage | 0.84 ^***^ | 0.09 | **<.001** | 0.92 ^***^ | 0.09 | **<.001** |
| Remorse | 0.27 | 0.69 | .70 | 0.17 | 0.67 | .79 |
| Repair | -0.47 | 0.73 | .52 | -0.53 | 0.70 | .44 |
| Responsibility | 0.18 | 0.71 | .79 | -0.13 | 0.68 | .85 |
|  |  |  |  |  |  |  |
| Physical Damage * Remorse | 0.02 | 0.12 | .89 | 0.10 | 0.11 | .36 |
| Physical Damage * Repair | -0.08 | 0.12 | .48 | -0.06 | 0.11 | .56 |
| Physical Damage * Responsibility | 0.03 | 0.11 | .79 | 0.10 | 0.11 | .36 |
| Emotional Damage * Remorse | -0.06 | 0.14 | .67 | -0.13 | 0.13 | .32 |
| Emotional Damage * Repair | 0.13 | 0.13 | .35 | 0.12 | 0.13 | .35 |
| Emotional Damage * Responsibility | -0.04 | 0.14 | .79 | -0.05 | 0.13 | .69 |
| **Random Effects** | | | | | | |
| σ^2^ |  | | | 4.19 | | |
| τ_00_ |  | | | 0.97 _ID_ | | |
| ICC |  | | | 0.19 | | |
| N |  | | | 395 _ID_ | | |
| R^2^ / R^2^ adjusted | 0.48 / 0.48 | | |  | | |
| R^2^ marginal/ R^2^ conditional | | | | 0.48 / 0.58 | | |
| Deviance | 7062.90 | | | 6995.08 | | |
| AIC | 7088.88 | | | 7023.10 | | |
| **** p<0.001* | | | | | | |

**Study 3:**

We compared Model 2b, a model that included random intercepts for each participant, with Model 1b, a general linear model without random intercepts.

| ***Table S9.*** *Detailed model parameters, Study 3.* | | | | | | |
| --- | --- | --- | --- | --- | --- | --- |
|  | **Model 1b: Linear Model** | | | **Model 2b: Mixed Effects Model** | | |
| *Predictors* | *Estimates* | *SE* | *p* | *Estimates* | *SE* | *p* |
| Intercept | -0.02 | 0.01 | .13 | -0.02 | 0.01 | .13 |
| Physical Damage | 0.44 ^***^ | 0.01 | **<.001** | 0.44 ^***^ | 0.01 | **<.001** |
| Emotional Damage | 0.31 ^***^ | 0.01 | **<.001** | 0.31 ^***^ | 0.01 | **<.001** |
| Remorse | 0.02 | 0.03 | .47 | 0.02 | 0.03 | .47 |
| Repair | -0.00 | 0.03 | .92 | -0.00 | 0.03 | .92 |
| Responsibility | 0.03 | 0.03 | .21 | 0.03 | 0.03 | .21 |
| Physical Damage * Remorse | 0.02 | 0.03 | .42 | 0.02 | 0.03 | .42 |
| Physical Damage * Repair | 0.00 | 0.03 | .90 | 0.00 | 0.03 | .90 |
| Physical Damage * Responsibility | 0.06 ^*^ | 0.03 | **.04** | 0.06 ^*^ | 0.03 | **.04** |
| Emotional Damage * Remorse | 0.00 | 0.03 | .87 | 0.00 | 0.03 | .87 |
| Emotional Damage * Repair | -0.02 | 0.03 | .59 | -0.02 | 0.03 | .59 |
| Emotional Damage * Responsibility | -0.03 | 0.03 | .33 | -0.03 | 0.03 | .33 |
| **Random Effects** | | | | | | |
| σ^2^ |  | | | 0.57 | | |
| τ_00_ |  | | | 0.00 _ID_ | | |
| N |  | | | 720_ID_ | | |
| R^2^ / R^2^ adjusted | 0.43 / 0.43 | | |  | | |
| R^2^ marginal/ R^2^ conditional | | | | 0.43 | | |
| Deviance | 8194.70 | | | 8194.70 | | |
| AIC | 8220.700 | | | 8222.70 | | |
| ** p<0.05 *** p<0.001* | | | | | | |

### Study 4, ROC curves

***Figure S5.*** *ROC Curve of the Performance of Study 2 Linear Regression Model Using 165 New Cases.*


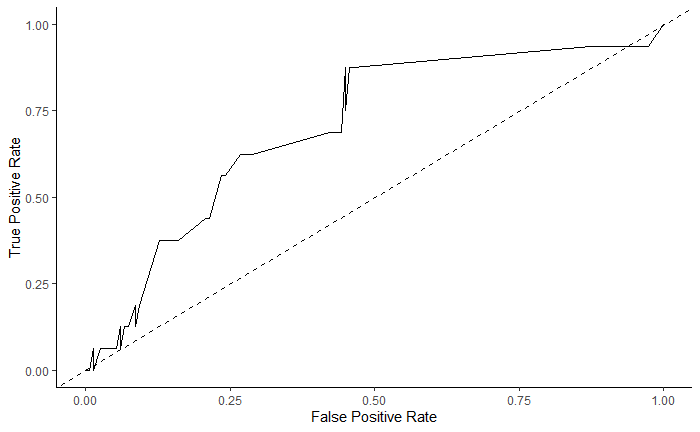


***Figure S6.*** *ROC Curve of the Performance of Study 3 Linear Regression Model Using 165 New Cases.*


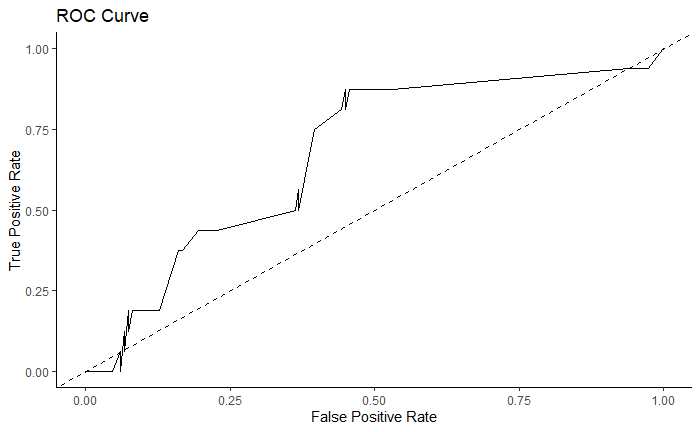


### Study 4, Additional Robustness Checks

In addition to the models predicting users’ intention to sue on the whole dataset consisting of 3,815 posts, we conducted additional models on smaller datasets based on different conditions to gain a deeper understanding of the predictive abilities of this phenomenon. The feature selection, modeling and validation were carried out in the same manner as the models reported in the main work, employing SelectKBest for feature selection, a random forest classifier and cross-validation. To avoid overfitting, we restricted the number of features as 10% of the length of the dataset.

#### Predicting Intention to Sue When Comments Refer to Legal Intentions.

We aimed to understand whether posts where comments address legal intentions could enhance the prediction performance. Our hypothesis was that examining comments that suggest the author may conduct a lawsuit or, conversely, suggest avoiding it, might yield better predictive power, indicating that others’ advice and comments influence the decision-making process.

To explore this, we filtered the dataset to include only cases where the comments contained specific stemmed terms related to the legal field ("lawyer", "lawsuit", "conduct", "suing", "prosecu", "attorney", "malp", "second opinion", "contact", "negl", "legal", "appeal", "plead", "charge"). The resulting dataset comprised 442 cases in total, with 398 cases representing no intention to sue (sue = 0) and 44 cases representing an intention to sue (sue = 1). 40 features were selected, with 34 features attributed to comments and only 6 features attributed to posts.

Figure S7 presents the mean ROC AUC of 0.75 received, indicating that we can achieve suitable prediction performance. The model’s performance is higher than that of the random forest model on the whole dataset, suggesting that when we consider the content of comments addressing the legal subject, comment- related features become the most influential predictors and contribute to higher prediction performance.

***Figure S7.*** *ROC Curve of the Performance of Random Forest Classifier Using Features of 422 Posts and Comments.*


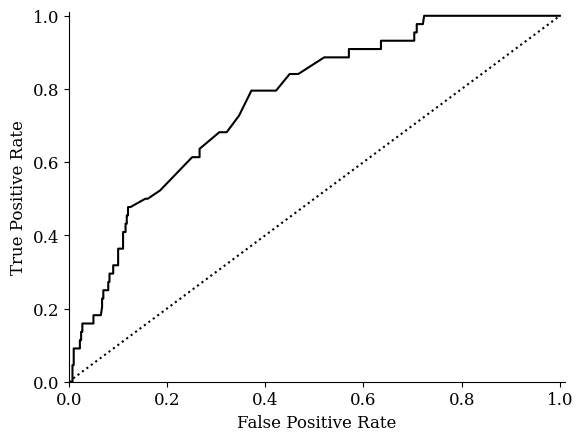


*Predicting Intention to Sue on Subset of Posts That are Similar to Each Other.*

Considering that our initial dataset’s classes were imbalanced, with posts representing an intention to sue consisting only 1.4% of the data and recognizing that our filtering method provided insights but still included some irrelevant posts, we sought to assess our prediction ability on a subset the contains all the posts representing the intention to sue (sue = 1) and posts that perceived as similar to them. Cosine similarity is a mathematical measure used to determine the similarity between two vectors (in our case, two posts). It ranges between -1 and 1, where 1 indicates that the two posts are identical, -1 indicates that they are diametrically opposed and 0 represents no similarity.

We employed cosine similarity to find posts from class 0 (sue = 0) that receive a similarity score of 0.5 or higher between them and at least one post from the other class (sue=1). The filtering process resulted in a dataset of 588 posts in total, with 55 posts representing an intention to sue and 533 representing no intention to sue. Using SelectKBest we selected the 50 best features, 45 of them being related to post attributes and five related to comment attributes.

Figure S8 presents the mean ROC AUC of 0.65 received, indicating that we can achieve high prediction performance. This model, representing a more concise and cleaner dataset that better reflects posts describing medical malpractice cases, along with its higher performance compared to the random forest model on the whole dataset, provides additional support for the predictive power of machine learning models using Reddit’s content to learn about people’s legal intentions.

***Figure S8.*** *ROC Curve of the Performance of Random Forest Classifier Using Features of 588 Similar Posts.*


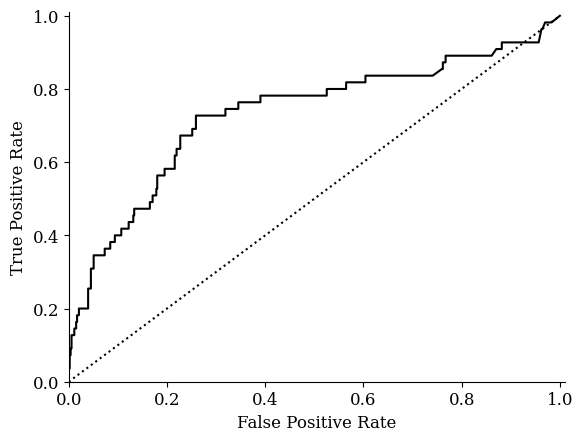


### References

Douglas, B. D., Ewell, P. J., & Brauer, M. (2023). Data quality in online human-subjects research: Comparisons between MTurk, Prolific, CloudResearch, Qualtrics, and SONA. PLOS ONE, 18(3), e0279720. https://doi.org/10.1371/JOURNAL.PONE.0279720

Peer, E., Rothschild, D., Gordon, A., Evernden, Z., & Damer, E. (2022). Data quality of platforms and panels for online behavioral research. Behavior Research Methods, 54(4), 1643–1662. https://doi.org/10.3758/S13428-021-01694-3/TABLES/13

Robbennolt, J. K. (2003). Apologies and legal settlement: An empirical examination. Michigan Law Review, 102(3), 460–516. https://doi.org/10.2307/3595367

RStudio Team. (2021). RStudio: Integrated development environment for R (v4.1.1). RStudio, PBC.

Turner, H., & Firth, D. (2012). Bradley-Terry models in R: The BradleyTerry2 package. Journal of Statistical Software, 48, 1–21. https://doi.org/10.18637/JSS.V048.I09
